# Coagulation Status in Dogs Naturally Infected with *Angiostrongylus vasorum*

**DOI:** 10.3390/pathogens10091077

**Published:** 2021-08-25

**Authors:** Nadja E. Sigrist, Lucienne Tritten, Claudia Kümmerle-Fraune, Natalie Hofer-Inteeworn, Rahel Jud Schefer, Manuela Schnyder, Annette P. N. Kutter

**Affiliations:** 1 Department for Small Animals, Vetsuisse Faculty, University of Zurich, 8057 Zurich, Switzerland; 2 Institute of Parasitology, University of Zurich, 8057 Zurich, Switzerland; lucienne.tritten@uzh.ch (L.T.); manuela.schnyder@uzh.ch (M.S.); 3 Department for Small Animals, Clinic for Small Animal Medicine, Vetsuisse Faculty, University of Zurich, 8057 Zurich, Switzerland; ckuemmerle@vetclinics.uzh.ch (C.K.-F.); nhofer@vetclinics.uzh.ch (N.H.-I.); 4 Department for Small Animals, Division of Emergency and Critical Care, Vetsuisse Faculty, University of Zurich, 8057 Zurich, Switzerland; rjud@vetclinics.uzh.ch; 5 Department for Clinical Diagnostics and Services, Section Anaesthesiology, Vetsuisse Faculty, University of Zurich, 8057 Zurich, Switzerland; akutter@vetclinics.uzh.ch

**Keywords:** angiostrongylosis, bleeding diathesis, fibrinogen

## Abstract

*Angiostrongylus vasorum* infection has been associated with coagulopathies including hyperfibrinolysis. We compared coagulation status including thromboelastometry (ROTEM) parameters in dogs naturally infected with *A. vasorum* versus healthy dogs to determine clinicopathological parameters associated with bleeding, hypocoagulopathy, and hyperfibrinolysis. Clinical signs, white blood cell count, platelet count, hematocrit, plasmatic coagulation tests (PT, aPTT, fibrinogen concentration), D-dimer, and ROTEM S parameters (Ex-tem, In-tem, Fib-tem, Ap-tem) were analysed and compared between bleeding, nonbleeding, and control dogs and between hypo- and normocoagulable animals. Clinical signs of bleeding were present in 6/9 (67%) hypocoagulable and 1/9 (11%) normocoagulable dogs. PT, fibrinogen concentration, and several ROTEM parameters were significantly different between hypocoagulable and normocoagulabe *A. vasorum* infected dogs. Hyperfibrinolysis was identified in 44% of infected dogs and was significantly more common in bleeding and hypocoagulable dogs. Hyperfibrinolysis was significantly associated with low MCFFib-tem but not with low fibrinogen concentration or increased D-dimers. CFTEx-tem > 248 swas 100% sensitive and 89% specific to predict hyperfibrinolysis. Hyperfibrinolysis, hypocoagulability and bleeding are common in *A. vasorum* infected dogs. Only Ex-tem and Fib-tem parameters and potentially PT were associated with bleeding or hypocoagulability. Ex-tem analysis enables detection of bleeding, hypocoagulability and hyperfibrinolysis within minutes.

## 1. Introduction

The canine heart- and lungworm *Angiostrongylus vasorum* (Baillet 1866) causes serious infections in dogs and is associated with a high mortality rate if not diagnosed and treated in due time [1]. The disease often causes a broad spectrum of clinical signs, including unspecific clinical signs. Bleeding diathesis, present in about 60% of clinical cases, can lead to rapid life-threatening complications [2] and may occur within the central nervous system, the lung, body cavities, mucous membranes, or in the integument [3,4]. The mechanisms and pathogenesis of these coagulopathies are poorly understood and several unresolved hypotheses such as chronic disseminated intravascular coagulation (DIC), FV and FVIII deficiency, immune-mediated thrombocytopenia, anticoagulants secreted by the parasite or activation of the fibrinolytic system of the host have been formulated [2,4,5,6,7,8,9]. A recent study evaluating serum proteome of experimentally infected dogs did not support an underlying DIC but rather consumption of some key factors of the coagulation and complement cascade [10]. Interestingly, a previous retrospective study identified hyperfibrinolysis (HFL) and associated hypofibrinogenemia in 67% of dogs with an *A. vasorum* infection and simultaneous clinical signs of bleeding [6]. Hyperfibrinolysis, which can be diagnosed with rotational thromboelastometry (ROTEM) [11], but not with plasmatic coagulation tests, may explain the bleeding diathesis seen in some dogs with *A. vasorum* infection [6].

Eventually, the identification of dogs at risk for bleeding diathesis remains a challenge. In a previous study, 73% of dogs with *A. vasorum* infection were hypocoagulable based on thromboelastography (TEG, which is comparable to ROTEM), with TEG parameters being significantly different between bleeding and nonbleeding dogs [2]. These authors found no association between other coagulation parameters (fibrinogen concentration, antithrombin activity, D-dimers, von Willebrand factor) and hypocoagulable parameters on TEG. 

To our knowledge, clinical signs of bleeding, plasmatic coagulation times, true prevalence of HFL and its association with bleeding diathesis had not been prospectively evaluated in dogs with *A. vasorum* infection. 

The present study aimed to describe the coagulation status in dogs naturally infected with *A.* *vasorum,* to establish the incidence of HFL and to determine clinicopathological parameters indicating the presence of HFL by comparing coagulation parameters of infected dogs to a healthy control group. An additional objective was to evaluate clinicopathological differences between hypocoagulable and normocoagulable *A. vasorum* infected dogs. 

We hypothesized that dogs naturally infected with *A. vasorum* and with clinical signs of bleeding would show changes in coagulation parameters, and that ROTEM parameters would be superior predictors of HFL than plasmatic coagulation tests or clinical signs at presentation.

## 2. Results

*Angiostrongylus vasorum* positive dogs included five Beagles, three mixed breed dogs and one each of the following breeds: Belgian Shepherd, Bernese Mountain Dog, Chihuahua, Dalmatian, Doberman, German Shepherd, French Bulldog, Golden Retriever, Hovawart, and Schnauzer. The control group included three mixed breed dogs and one Beagle, one Belgian Shepherd, one German Pointer, one Golden Retriever, and one Nova Scotia Duck Tolling Retriever. Breed (*p* = 0.483), sex (*p* = 0.230), age (*p* = 0.154), and weight (*p* = 0.441) were not significantly different between dogs with *A. vasorum* infection and control dogs. 

Of the 18 dogs with *A. vasorum* infection, 7 presented with clinical or magnetic resonance imaging (MRI) signs of bleeding (39%). Bleeding occurred in the brain (*n* = 3), episcleral (*n* = 2), lip (*n* = 2), lung (*n* = 1), and nose (*n* = 1). Three of seven dogs showed more than one bleeding location. All three dogs with signs of central nervous system (CNS) bleeding on MRI presented with episcleral bleeding. 

Nine of 18 (50%) infected dogs were hypocoagulable. Six of 7 (86%) bleeding dogs and 3/11 (27%) of nonbleeding dogs were hypocoagulable (*p* = 0.050) while clinical signs of bleeding were present in 6/9 (67%) hypocoagulable and 1/9 (11%) normocoagulable dogs (*p* = 0.050). Hypocoagulable dogs exhibited a median of four different hypocoagulable parameters (2–7). 

Differences between control, infected bleeding and infected nonbleeding dogs are summarized in Table 1 and Table 2. None of the evaluated clinical parameters at presentation was significantly different between groups (Table 1). The group of dogs with bleeding diathesis was thrombocytopenic, had a prolonged prothrombin time (PT) and hypocoagulable Ex-tem and Fib-tem parameters compared to both nonbleeding and control dogs. 

Differences between hypocoagulable and normocoagulable *A. vasorum* infected dogs are summarized in Table 3 and Table 4. None of the clinical signs occurred in different frequency between hypo- and normocoagulable dogs. Platelet count (but not the presence of thrombocytopenia), PT, and several ROTEM parameters were significantly different between hypocoagulable and normocoagulabe *A. vasorum* infected dogs. Clinical signs and the localization of clinical manifestations were not significantly different between hypo- and normocoagulable dogs. 

Overall incidence of HFL defined by maximum lysis above the reference interval in any of the ROTEM tracings was 44% in dogs with *A. vasorum* infection (8/18 dogs) and was significantly higher in bleeding dogs (*p* = 0.011; Table 2) and hypocoagulable dogs (*p* = 0.015, Table 4). Four of 7 (57%) bleeding dogs showed increased Ex-tem lysis compared to none of the nonbleeding dogs (*p* = 0.011). Hyperfibrinolysis was significantly associated with low MCF_Fib-tem_ (*p* = 0.001) but not with low fibrinogen concentration (*p* = 0.088). AUROC results for prediction of HFL are shown in Table 5. CFT_Ex-tem_ > 248 s was 100% sensitive and 89% specific and CT_Extem_ > 103 s was 75% sensitive and 99% specific. Hyperfibrinolysis was not associated with high D-dimers (*p* = 0.559). 

All dogs were discharged from the hospital. Four dogs had been treated with fresh frozen plasma (20–30 mL/kg BW) and additionally received tranexamic acid (20–60 mg/kg). These four dogs were bleeding, hypocoagulable, and lacked clot formation in the Fib-tem tracing. An additional bleeding dog was treated with tranexamic acid. Plasma transfusion was significantly associated with low fibrinogen Clauss (*p* = 0.045), low MCF_Fib-tem_ (*p* = 0.011), CT_Ex-tem_ prolongation (*p* = 0.011), and bleeding (*p* = 0.011) but not with hypocoagulability (*p* = 0.082). 

Ap-tem tracings showed both weaker and stronger MCFs than Ex-tem profiles. MCF_Ap-tem_ was not stronger (indicating HFL) in any of the 5 dogs with HFL diagnosed in Ex-tem or In-tem but was > 15% stronger in 4/15 dogs with no HFL and ML’s within the reference interval.

## 3. Discussion

The present prospective study identified bleeding diathesis in 39% of dogs naturally infected with *A. vasorum*. Among those, 86% were diagnosed hypocoagulable by thromboelastometry and plasmatic coagulation tests. Hyperfibrinolysis was present in 44% of all infected dogs and in 56% of those dogs with bleeding diathesis. 

The true incidence of *A. vasorum* associated bleeding diathesis remains unknown, as this study was based mostly on referred cases. However, our incidence of 39% is in the range of previously reported incidences between 35% and 57% [6,12]. 

The main clinical signs of dogs naturally infected with *A. vasorum* are coughing and tachypnea/dyspnea together with unspecific signs such as lethargy, anorexia, and vomiting/diarrhea [2,4,12,13,14,15]. Haemorrhage and neurological signs are less frequently observed [4,12]. None of the clinically evaluated parameters in the current study were significantly associated with the presence of haemorrhage, as previously observed [2].

ROTEM analysis identified several parameters that were significantly different between dogs with bleeding diathesis compared to infected dogs without bleeding and healthy control dogs. Prolonged CT_Ex-TEM_, CFT_Ex-TEM_, decreased MCF_Ex-tem_ and MCF_Fib-tem_, and Ex-TEM HFL were significantly associated with the presence of bleeding. These findings indicate that both clot formation and clot strength are affected in dogs with *A. vasorum* induced coagulopathy. 

In contrast to a previous report [2], our study population of dogs with bleeding diathesis showed a significantly lower median platelet count compared to both nonbleeding dogs and healthy control dogs. The platelet count of bleeding dogs was below the reference interval but not in a range that would explain spontaneous bleeding. Several studies and case reports demonstrated thrombocytopenia and prolonged clotting times [8,16,17,18], but experimental studies indicate that such findings are inconsistent over time [7,9,13]. Thrombocytopenia was significantly associated with the presence of bleeding in our study population and 83% of dogs with bleeding were thrombocytopenic (*n* = 5/6), while this was observed in only 10% (*n* = 1/10) of nonbleeding infected dogs. However, no reasonable platelet count cut-off value to identify dogs with bleeding diathesis could be identified and whether the decreased platelet count is the cause or the result of bleeding is unknown. Together with the identified mild thrombocytopenia, we interpret this finding as evidence that thrombocytopenia is a consequence rather than the cause of bleeding, indicating that mechanisms other than thrombocytopenia are causing bleeding diathesis. 

The PT, aPTT, and fibrinogen concentration were not significantly associated with bleeding, indicating that viscoelastic testing is superior to classical plasmatic coagulation times for the identification of bleeding in *A. vasorum* infected dogs. 

While overt clinical signs of bleeding will guide the clinician to investigate the coagulation status, internal bleeding in the brain or lung may be fatal if hypocoagulability is not detected and treated [17,19]. The recognition of dogs at risk for bleeding diathesis remains a challenge [6]. Therefore, the identification of valid prediction parameters to detect hypocoagulability are of relevant clinical interest. 

We therefore analysed clinical and laboratory parameters to detect hypocoagulability in *A. vasorum* infected dogs. Of interest, only two-thirds of hypocoagulable dogs showed clinical signs of bleeding and more than one-quarter of nonbleeding dogs were hypocoagulable and hence at risk to develop bleeding during hospitalization. Cough, pulmonary changes consistent with radiographic signs of *A. vasorum* infection [20] on radiographs and presence of respiratory signs were not associated with hypocoagulability, suggesting that respiratory signs and the typical interstitial-alveolar pattern seen on thoracic radiographs are not caused by haemorrhage; consequently, thoracic radiographs will not aid in the identification of hypocoagulable dogs. This finding is supported by a study evaluating radiographic, CT and necropsy findings in experimentally infected dogs and linking them to granulomatous inflammation rather than haemorrhage [20].

Of note, all dogs with neurological signs were hypocoagulable. Such *A. vasorum* infected dogs commonly do not present with classical pulmonary signs and internal haemorrhage may not be apparent [17]. However, the three dogs with neurological signs all presented with episcleral bleeding, a finding that should be investigated in a larger study population. Until then, it is important to not only include *A. vasorum* infection as a differential diagnosis to central nervous clinical signs but to further investigate the coagulation status for hypocoagulability if the dogs are infected. 

In contrast, none of the dogs with an incidental diagnosis of *A. vasorum* infection was hypocoagulable. We suspect that these dogs were either not infected long enough to develop coagulopathies or with too few worms to show hypocoagulability or clinical signs of bleeding. Experimentally inoculated dogs with higher worm burdens showed earlier and more severe respiratory signs and more pronounced coagulopathies [13]. The magnitude of parasite burden was unknown in our study population; therefore, a direct comparison cannot be made. In addition, individual differences between dogs cannot be excluded: the study of dog serum proteome in experimentally infected dogs revealed that the lectin pathway and the coagulation cascades were particularly affected; these may be additionally associated with individual differences that altogether may explain the onset or absence of hypocoagulability [10]. 

In the present work, not all hypocoagulable dogs exhibited identifiable signs of bleeding, highlighting the need for laboratory analysis of coagulation status to identify dogs at risk of bleeding. Hypocoagulable dogs had significantly longer PT and abnormal Ex-tem, In-tem, and Fib-tem parameters but no significant thrombocytopenia or prolonged aPTT. Previously, no associations could be identified between coagulation parameters (fibrinogen concentration, antithrombin activity, D-dimers, von Willebrand factor) and hypocoagulable thromboelastography parameters [2], indicating that viscoelastic tests may be more appropriate to identify hypocoagulable dogs. 

We recently retrospectively identified HFL and associated hypofibrinogenemia in *A. vasorum* infected dogs [6], two findings that are supported by the data presented here. Hyperfibrinolysis was identified in 44% of dogs naturally infected with *A. vasorum* and 5/9 (56%) of bleeding dogs showed increased Ex-tem or In-tem lysis compared to 1/9 (11%) nonbleeding dogs. Both bleeding and hypocoagulable dogs had low to no fibrin clot formation compared to nonbleeding dogs. Hypofibrinogenemia was significantly more common in hypocoagulable dogs. Bleeding dogs displaying decreased fibrinogen concentration have also been described previously [2]. As suggested earlier [5,6], the cause of decreased fibrin clot formation in the Fib-tem tracing may be explained by HFL and not by low fibrinogen concentrations. Hyperfibrinolysis was significantly associated with MCF_Fib-TEM_ but not with fibrinogen concentration measured by Clauss. Identification and treatment of HFL therefore seems to be of clinical relevance. Neither platelet count nor plasmatic coagulation times are able to predict or diagnose HFL. ROTEM analysis; however, was able to predict HFL by means of prolonged CT and CFT within minutes. 

The initial study protocol envisaged Ap-tem profiles to confirm HFL identified in Ex-tem profiles. Our study, showing inconsistent Ap-tem results, supports earlier evidence that Ap-tem assays are not helpful in the diagnosis of HFL in dogs [6,11]. 

Elevated D-dimer concentrations in dogs with *A. vasorum* infection have also been described [2,17,21]. Although expected to increase with fibrinolysis, D-dimer levels were not different between hypo- and normocoagulable dogs in our study population and were not associated with HFL. A reason for this finding may be that the presence of D-dimers requires lysis of crosslinked fibrin while *A. vasorum* associated lysis of fibrinogen or fibrin strands may occur prior to crosslinking to a stable fibrin clot [6]. Fibrinogen, being an acute phase protein, is expected to be present at high levels in dogs with *A. vasorum* infections. Another possible explanation for the lack of stable fibrinogen clot is FXIII deficiency or malfunction. FXIII is needed for cross-linking of fibrin and congenital or acquired deficiency leads to unexpected bleeding in people [22,23]. In line with this, a recent time-course proteomics study performed on sera of *A. vasorum* infected dogs identified FXIII-B, a subunit of FXIII, as downregulated among other candidates of the coagulation cascade and complement pathway compared to before inoculation with the parasite [10]. 

While significantly more hypocoagulable dogs were treated with tranexamic acid, hypocoagulability was not significantly associated with administration of a plasma transfusion. Significantly more plasma transfusions were given to bleeding dogs, however, a plasma transfusion was not deemed necessary in 3/7 dogs with bleeding, based on coagulation parameters. Together with the wide variety of identified ROTEM abnormalities in hypocoagulable dogs, this indicates that coagulation testing the complete set of coagulation parameters is crucial to identify not only dogs at risk for bleeding but also to treat the coagulation disorder with specific therapeutic measures. 

In former studies up to 30% of *A. vasorum* infected dogs died [2,12], while all dogs in this study were discharged from the hospital. It is possible that early identification of coagulation problems and early therapeutic intervention has led to the excellent survival rate in our study population. As all patients were treated based on ROTEM results as well as at the discretion of the clinician in charge, the effect of treatment on survival cannot be investigated further. 

This study included only a small group of *A. vasorum* infected dogs and only part of them showed bleeding diathesis. Unfortunately, bleeding dogs were often presented during emergency hours when plasmatic coagulation testing was not available. We however showed that results of plasmatic coagulation testing should be interpreted with caution. Additionally, when ROTEM tests were chosen by the clinician in charge during emergency service hours, in several patients In-tem analysis was not performed. In-tem parameters between bleeding and nonbleeding *A. vasorum* infected dogs could therefore not be compared. Bleeding was furthermore based on visible clinical signs of bleeding and MRI, with the possibility of having falsely assigned an internally bleeding dog to the nonbleeding group.

## 4. Materials and Methods

Dogs presenting to the Small Animal Clinic, Vetsuisse Faculty, University of Zurich between March 2016 and July 2019 and being diagnosed with *A. vasorum* infection by either serological ELISA antigen or rapid-assay (AngioDetect^TM^) [24,25] and/or copromicroscopic identification of first stage larvae by the Baermann funnel method were eligible to enter this and a concurrent study. Healthy dogs were used as a control group. Control dogs had a normal complete blood cell and chemistry panel, plasmatic coagulation times including fibrinogen measured by Clauss and were negative for *A. vasorum* infection. The project was approved by the ethics committee on animal research of the canton of Zurich (ZH001/16,27384) and informed owner consent was obtained for both healthy and infected dogs, in which additional blood tests were performed for the study. 

Dogs were excluded if they were pre-treated with antifibrinolytics, blood products or NSAIDS within five days of presentation and if ROTEM analysis was not performed within 30 min after blood sampling. 

Signalment, sex, weight, presenting complaint, clinical signs including the presence of bleeding were determined at presentation. The infected dogs were assigned to the bleeding or the nonbleeding group based on the presence of clinical signs of bleeding or MRI signs compatible with bleeding in dogs undergoing MRI exam. In addition, clinical signs were categorized as mucocutaneous bleeding, lung or CNS localization or incidental diagnosis without clinical signs (localization of clinical manifestation). Diagnostic imaging findings and survival to discharge were extracted later from patient records. 

Approximately 8–12 mL of venous blood were drawn at presentation or immediately after diagnosis of *A. vasorum* infection using a 20 G hypodermic needle. Blood samples were transferred to tubes in the following order: two 3.2% citrate and one EDTA tube (all Sarstedt AG, Sevelen, Switzerland). One citrate and the EDTA tube were sent to the in-house laboratory for plasmatic coagulation tests (PT, aPTT, TT, fibrinogen_Clauss_, and D-dimers) (Start 4 STAGO CH SA, Glattbrugg, Switzerland) and complete blood cell status (Sysmex-XT 2000iV, Sysmex Cooperation, Kobe, Japan). Another citrate tube was placed within the warming plate of the ROTEM device for 10–20 min. 

Ex-tem S, In-tem S, Fib-tem S, and Ap-tem S analysis (TEM International GmbH, Munich) were performed by the primary investigators according to an institutional protocol based on the manufacturer’s instruction and international guidelines [26,27]. Briefly, 300 µL citrated whole blood were incubated with the lyophilized reagent before placement in the cup and start of measurement. An automated pipetting system was used. Tracings were run for 60 min. All tracings were checked for artefacts by two of the authors (N.E.S, A.P.N.K). Clotting time (CT), clot formation time (CFT), alpha angle (a), maximum clot firmness (MCF), maximum clot elasticity (MCE) and maximum lysis (ML) were extracted from the ROTEM database. A green line in the fib-tem tracing was defined as an MCF_Fib-tem_ of 0 mm. If MCF did not reach 20 mm, CFT was defined as 3600 s. Hypofibrinogenemia was defined as fibrinogen measured by Clauss < 1.2 g/L (in-house reference interval) or MCF_Fib-tem_ < 2 mm. 

Based on ROTEM profiles, dogs were categorized as hypocoagulable if ≥ 2 of the following parameters were hypocoagulable compared to reference intervals [28]: CT_Ex-tem_ or PT, CT_In-tem_ or aPTT, CFT_Ex-tem / In-tem_ or α _Ex-tem / In-tem_, MCF_Ex-tem / In-tem / Fib-tem_ or MCE_Ex-tem / In-tem / Fib-tem_. Hyperfibrinolysis was defined as ML > reference interval in any of the tracings. Dogs were treated at the discretion of the clinician in charge, generally based on a standardized protocol including 50 mg/kg BW fenbendazole daily for three weeks and 1 mg/kg BW prednisolone daily for seven days was administered; strict exercise constraint was advised. Fresh frozen plasma (20 mL/kg BW) was transfused for hypofibrinogenemia and tranexamic acid was prescribed to treat HFL (20 mg/kg BW, repeated until HFL resolved) as needed. 

### Statistical Analysis 

Data were entered into a spreadsheet and analyses were performed using the statistical software program IBM SPSS (Version 23.0. IBM Corp, Armonk, NY, USA). Continuous data is presented as median (min-max). Clinical signs and coagulation parameters were compared between dogs with and without bleeding diathesis and control dogs using Kruskal–Wallis tests followed by post hoc analysis with Dunn-Bonferroni for continuous variables and chi^2^ test for categorical variables. In a second step dogs with *A. vasorum* infection were grouped as hypo- or normocoagulable and clinical signs and coagulation parameters were compared across groups using Mann–Whitney U-test or Fisher’s exact test. The accuracy of standard coagulation tests and ROTEM parameters to determine dogs with HFL was evaluated using receiver operating characteristics (ROC) and the area under the ROC curve (AUROC). Youden’s J statistic was used to select the optimum cut-off point of the ROC curves. A value of *p* < 0.05 was considered significant.

## 5. Conclusions

In conclusion, HFL, hypocoagulability and bleeding are commonly occurring in *A. vasorum* infected dogs. None of the evaluated clinical parameters are significantly associated with bleeding or hypocoagulability, and plasmatic coagulation tests did not allow clear discrimination between bleeding and nonbleeding *A. vasorum* infected dogs. In contrast, most ROTEM parameters were significantly different between groups, allowing the differentiation between bleeding and nonbleeding *A. vasorum* infected dogs. Ex-tem analysis was able to detect bleeding, hypocoagulability, and HFL within a few minutes after blood draw and may therefore be recommended.

## Figures and Tables

**Table 1:** Haematological, plasmatic coagulation, and ROTEM parameters in 8 control dogs and 18 dogs with *A. vasorum* infection with and without signs of bleeding

		Groups									Comparison between Groups			
Parameter	Reference Interval	Control (*n* = 8)			*A. vasorum* Infected Nonbleeding (*n* = 11)			*A. vasorum* Infected Bleeding (*n* = 7)			All Groups	Control vs. Nonbleeding	Control vs. Bleeding	Bleeding vs. Nonbleeding
		n/N	Median	Range	n/N	Median	Range	n/N	Median	Range	*p* *	P^+^	P^+^	P^+^
Haematological and plasmatic coagulation results														
Haematocrit (%)	42–55	8/8	48	39–51	11/11	42	36–52	6/7	41	32–46	0.064			
White blood cells (10 × 9/L)	4.7–11.3	8/8	7.7	5.1–10.7	11/11	11.7	9.2–21.1	6/7	13.5	10.9–18	0.001	0.003	0.002	1.000
Platelet count (10 × 9/L)	150–399	7/8	275	213–403	10/11	280	81–421	6/7	130	87–174	0.014	1.000	0.030	0.023
PT (s)	6.5–8.7	8/8	7.2	6.3–7.8	9/11	7.7	7.0–9.9	2/7	17.3	10.5–24.1	0.010	0.126	0.015	0.351
aPTT (s)	10.2–13.8	8/8	11.8	9.6–14.2	9/11	12.1	10.0–15.5	2/7	20.4	14.1–26.7	0.091	NA	NA	NA
Fibrinogen Clauss (g/L)	1.2–2.8	8/8	1.9	1–2.3	9/11	1.9	0.7–2.8	3/7	0.9	0.1–1.2	0.052	NA	NA	NA
D-dimer (mg/dL)	<0.3	8/8	0.1	0.1–1.7	8/11	0.7	0.1–3.8	2/7	1.4	0.9–1.8	0.123	NA	NA	NA
ROTEM parameters														
CT_Ex-tem_ (s)	23–87	8/8	34	25–94	11/11	29	24–113	7/7	138	80–251	0.001	1.000	0.012	0.001
CFT_Ex-tem_ (s)	85–357	8/8	188	82–264	11/11	180	53–541	7/7	411	210–3600	0.008	1.000	0.017	0.019
Alpha_Ex-tem_ (°)	42-77	8/8	58	52–74	11/11	58	35–83	7/7	41	18–53	0.011	1.000	0.019	0.029
MCF_Ex-tem_ (mm)	32-65	8/8	50	41–66	11/11	50	28–72	7/7	29	14-47	0.008	1.000	0.020	0.014
MCE_Ex-tem_	45–142	8/8	100	70–197	11/11	99	38–254	7/7	41	17–89	0.007	1.000	0.019	0.013
ML_Ex-tem_ (%)	0–12	8/8	5	1–9	11/11	3	0–11	7/7	32	0-100	0.079	NA	NA	NA
CT_In-tem_ (s)	133–210	8/8	160	151–206	10/11	184	149–258	1/7	(289)	NA	0.116	NA	NA	NA
CFT_In-tem_ (s)	59.201	8/8	102	59–121	10/11	84	51–289	1/7	(294)	NA	0.207	NA	NA	NA
Alpha_In-tem_ (°)	58–78	8/8	71	67–78	10/11	73	47–79	1/7	(48)	NA	0.279	NA	NA	NA
MCF_In-tem_ (mm)	52–71	8/8	60	57–68	10/11	63	40–72	1/7	(41)	NA	0.235	NA	NA	NA
MCE_In-tem_	108–242	8/8	153	130–211	10/11	169	67–260	1/7	(69)	NA	0.197	NA	NA	NA
ML_In-tem_ (%)	0-3	8/8	1	0–5	10/11	0	0–80	1/7	(0)	NA	0.568	NA	NA	NA
CT_Fib-tem_ (s)	21–112	8/8	33	22–464	10/11	36	23–3600	7/7	3600	278–3600	0.002	1.000	0.006	0.005
MCF_Fib-tem_ (mm)	2–9	8/8	4	2–8	11/11	6	0–14	7/7	0	0–2	0.001	1.000	0.028	0.001
MCE_Fib-tem_	2–9	8/8	4	3–9	11/11	7	0–16	7/7	0	0–2	0.001	1.000	0.024	0.001
CT_Ap-tem_ (s)	21–75	8/8	29	23–51	9/11	34	24–95	3/7	238	112–265	0.019	1.000	0.016	0.070
CFT_Ap-tem_ (s)	99–485	8/8	198	127–371	9/11	162	49–428	3/7	1044	476–3600	0.023	1.000	0.063	0.020
Alpha_Ap-tem_ (°)	42–79	8/8	66	41–78	9/11	59	43–78	3/7	23	17–36	0.023	1.000	0.021	0.056
MCF_Ap-tem_ (mm)	32–65	8/8	49	37–57	9/11	52	30–70	3/7	26	16–37	0.034	1.000	0.093	0.031
MCEAp-tem	47–175	8/8	98	59–132	7/11	96	43–231	3/7	34	19–59	0.034	0.980	0.085	0.033
ML _p-tem_ (%)	0–10	8/8	5	0–8	9/11	1	0–13	3/7	0	0–100	0.247	NA	NA	NA
Delta MCF_Ap-tem_ -MCF_Ex-tem_ (%)	NA	8/8	−14	−22–33	9/11	8	−9–71	3/7	4	−5–14	0.312	NA	NA	NA

*p* *, Kruskal–Wallis test; P**^+^**, post hoc analysis with Dunn–Bonferroni. aPTT, activated partial thromboplastin time; CT, clotting time; CFT, clot formation time; CT, clotting time; CFT, clot formation time; MCF, maximum clot firmness; ML, maximum lysis; NA, not assessed; PT, prothrombin time; MCE, maximum clot elasticity; MCF, maximum clot firmness; ML, maximum lysis; PT, prothrombin time.

**Table 2:** Respiratory clinical and radiographic signs, haematological and ROTEM abnormalities and treatment in 8 control, 11 infected nonbleeding, and 7 infected bleeding dogs with *A. vasorum* infection

	Groups			Comparison between Groups *			
Parameter	Control (*n* = 8)	*A. vasorum* Infected Nonbleeding (*n* = 11)	*A. vasorum* Infected Bleeding (*n* = 7)	All Groups	Control vs. Nonbleeding	Control vs. Bleeding	Bleeding vs. Nonbleeding
	n/N (%)	n/N (%)	n/N (%)	*p*	*p*	*p*	*p*
Respiratory clinical and radiographic signs indicative for canine angiostrongylosis							
Cough	0/8	5/11 (46%)	2/7 (28%)	0.063	NA	NA	NA
Respiratory distress	0/8	2/11 (18%)	1/7 (14%)	1.000	NA	NA	NA
Interstitial pattern on chest radiographs	NA	4/6 (67%)	4/4 (100%)	0.467	NA	NA	NA
Abnormal haematological and plasmatic coagulation parameters							
Thrombocytopenia (<150 × 10^9^/L)	1/8 (13%)	1/10 (10%)	5/6 (83%)	0.004	1.000	0.026	0.008
PT > 8.7 s	0/8	2/9 (22%)	2/2 (100%)	0.017	0.471	0.022	0.109
aPTT > 13.8 s	1/8 (13%)	1/9 (11%)	2/2 (100%)	0.053	1.000	0.067	0.055
Fibrinogen Clauss <1.2 g/L	0/8	1/9 (11%)	2/3 (67%)	0.046	1.000	0.055	0.127
D-dimers > 0.3 mg/dL	2/8 (25%)	4/8 (50%)	2/2 (100%)	0.171	0.608	0.133	0.467
Hypocoagulable ROTEM parameters							
CT_Ex-tem_ > 87 s	1/8 (13%)	1/11 (9%)	6/7 (86%)	0.001	1.000	0.010	0.002
CFT_Ex-tem_ > 328 s	0/8	3/11 (27%)	6/7 (86%)	0.002	0.228	0.001	0.050
Alpha_Ex-tem_ < 42°	0/8	1/11 (9%)	4/7 (57%)	0.011	1.000	0.026	0.047
MCF_Ex-tem_ < 32 mm	0/8	1/11 (9%)	5/7 (71%)	0.002	1.000	0.007	0.013
MCE_Ex-tem_ < 45	0/8	1/11 ()%)	5/7 (71%)	0.002	1.000	0.007	0.013
ML_Ex-tem_ > 12%	0/8	0/11	4/7 (57%)	0.002	NA	0.026	0.011
CT_In-tem_ > 211 s	0/8	3/10 (30%)	1/1 (100%)	0.063	0.216	0.111	0.364
CFT_In-tem_ > 130 s	0/8	2/10 (20%)	1/1 (100%)	0.075	0.477	0.111	0.273
Alpha_In-tem_ > 58°	0/8	1/10 (10%)	1/1 (100%)	0.105	1.000	0.111	0.182
MCF_In-tem_ < 57 mm	0/8	2/10 (20%)	1/1 (100%)	0.075	0.477	0.111	0.273
MCE_In-tem_ < 108	0/8	2/10 (20%)	1/1 (100%)	0.075	0.477	0.111	0.273
ML_In-tem_ > 5%	0/8	2/10 (20%)	0/1	0.532	0.477	NA	1.000
CT_Fib-tem_ > 112 s	3/8 (38%)	1/10 (10%)	7/7 (100%)	0.001	0.275	0.026	<0.001
MCF_Fib-tem_ 0 mm	0/8	1/11 (9%)	6/7 (86%)	<0.001	1.000	0.001	0.002
MCF_Ap-tem_ > MCF_Ex-tem_	2/8 (25%)	2/9 (18%)	0/3	1.000	1.000	1.000	1.000
Treatment							
Plasma transfusion	NA	0/11	4/7 (57%)	NA	NA	NA	0.011
Tranexamic acid	NA	0/11	5/7 (71%)	NA	NA	NA	0.002

* chi^2^ test; aPTT, activated partial thromboplastin time; CT, clotting time; CFT, clot formation time; AUC, area under the curve; CI, confidence interval; CT, clotting time; CFT, clot formation time; MCF, maximum clot firmness; ML, maximum lysis; PT, prothrombin time; MCE, maximum clot elasticity; MCF, maximum clot firmness; ML, maximum lysis; NA, not assessed; PT, prothrombin time.

**Table 3:** Differences in haematological, plasmatic coagulation and ROTEM parameters between hypo- vs. normocoagulable *A. vasorum* infected dogs

Parameter	Normocoagulable (*n* = 9)			Hypocoagulable (*n* = 9)					
	n/N	Median	Range	n/N	Median	Range	Reference Interval	*p* *	
Haematological and plasmatic coagulation parameters									
Haematocrit (%)	9/9	44	32–52	9/9	41	36–46	42–55	0.423	
White blood cells (10^9^/L)	9/9	11.7	9.2–15.2	8/9	13.7	10.9–21.1	4.7–11.3	0.139	
Platelet count (10^9^/L)	8/9	292	137–421	8/9	136	81–286	150–399	0.007	
PT (s)	6/9	7.6	7.0–8.5	5/9	9.9	7.7–24.1	6.5–8.7	0.017	
aPTT (s)	6/9	12.1	10.0–12.6	5/9	14.1	11.6–26.7	10.2 –13.8	0.082	
Fibrinogen Clauss (g/L)	7/9	1.9	1.2–2.8	5/9	0.9	0.1–2.3	1.2–2.8	0.073	
D-dimer (mg/dL)	6/9	0.7	0.1–3.8	4/9	0.9	0.5–1.8	<0.3	1.000	
ROTEM parameters									
CT_Ex-tem_ (s)	9/9	29	24–80	9/9	134	27–251	23–87	0.001	
CFT_Ex-tem_ (s)	9/9	175	53–359	9/9	411	180–3600	85–357	0.001	
Alpha_Ex-tem_ (°)	9/9	58	47–83	9/9	41	18–58	42–77	0.003	
MCF_Ex-tem_ (mm)	9/9	50	35–72	9/9	29	14–54	32–65	0.002	
MCE_Ex-tem_	9/9	99	53–254	9/9	41	17–117	45–142	0.002	
ML_Ex-tem_ (%)	9/9	2	0–4	9/9	11	0–100	0–12	0.063	
Ct_In-tem_ (s)	7/9	173	149–233	4/9	226	168–289	133–210	0.230	
CFT_In-tem_ (s)	7/9	83	51–105	4/9	255	79–294	59–201	0.073	
Alpha_In-tem_ (°)	7/9	73	69–79	4/9	56	47–74	58–78	0.073	
MCF_In-tem_ (mm)	7/9	63	62–72	4/9	44	40–64	52–71	0.073	
MCE_In-tem_	7/9	172	164–260	4/9	79	67–177	108–242	0.073	
ML_In-tem_ (%)	7/9	0	0–22	4/9	1	0–80	0–3	0.412	
CT_Fib-tem_ (s)	8/9	36	25–278	9/9	3600	23–3600	21–112	0.008	
MCF_Fib-tem_ (mm)	9/9	6	2–14	9/9	0	0–5	2–9	<0.001	
MCE_Fib-tem_	9/9	7	2–16	9/9	0	0–5	2–9	<0.001	
CT_Ap-tem_ (s)	6/9	32	24–45	6/9	104	26–265	21–75	0.041	
CFT_Ap-tem_ (s)	6/9	125	59–193	6/9	452	238–3600	99–485	0.002	
Alpha_Ap-tem_ (°)	6/9	67	55–78	6/9	40	17–52	42–79	0.002	
MCF_Ap-tem_ (mm)	6/9	57	49–70	6/9	34	16–49	32–65	0.002	
MCE_Ap-tem_	4/9	152	94–231	6/9	51	19–96	47–175	0.019	
ML_Ap-tem_ (%)	6/9	1	0–2	6/9	0	0–100	0–10	0.818	
Delta MCF_Ap-tem_-MCF_Ex-tem_ (%)	6/9	9.5	1.7–71.4	6/9	5.6	−9.3–14.3		0.240	

* *p* determined by Mann–Whitney U-test; aPTT, activated partial thromboplastin time; CT, clotting time; CFT, clot formation time; CT, clotting time; CFT, clot formation time; MCF, maximum clot firmness; ML, maximum lysis; PT, prothrombin time; MCE, maximum clot elasticity; MCF, maximum clot firmness; ML, maximum lysis; PT, prothrombin time.

**Table 4:** Comparison of the occurrence of clinicopathological variables between normo- and hypocoagulable *A. vasorum* infected dogs

Parameter	Normocoagulable (*n* = 9)	Hypocoagulable (*n* = 9)	
	n/N (%)	n/N (%)	*p*
Localization of clinical manifestation			
Mucocutaneous bleeding	1/9 (11%)	2/9 (22%)	0.086
Lung	4/9 (44%)	2/9 (22%)	
CNS	0	4/9 (44%)	
Incidental diagnosis	4/9 (44%)	1/9 (11%)	
Respiratory clinical and radiographic signs and bleeding indicative for canine angiostrongylosis			
Cough	4/9 (44%)	2/9 (22%)	0.620
Respiratory distress	1/9 (11%)	2/9 (22%)	0.500
Alveolar-interstitial pattern on chest radiographs	4/5 (80%)	4/5 (80%)	1.000
Clinical signs of bleeding	1/9 (11%)	6/9 (67%)	0.050
Hematological parameters correlated with coagulation			
Thrombocytopenia	1/8 (13%)	5/8 (63%)	0.119
PT > 8.7 s	0/6	4/5 (80%)	0.015
aPTT > 13.8 s	0/6	3/5 (60%)	0.061
Fibrinogen Clauss < 1.2 g/L	0/7	3/5 (60%)	0.045
D-dimer > 0.3 mg/dL	3/6 (50%)	3/4 (75%)	0.571
ROTEM parameters			
CT_Ex-tem_ > 87 s	0/9	7/9 (78%)	0.002
CFT_Ex-tem_ > 328 s	1/9 (11%)	8/9 (89%)	0.003
MCF_Ex-tem_ < 32 mm	0/9	6/9 (67%)	0.009
alpha_Ex-tem_ < 42°	0/9	5/9 (56%)	0.029
MCE_Ex-tem_ < 45	0/9	6/9 (67%)	0.009
ML_Ex-tem_ > 12%	0/9	4/9 (44%)	0.082
CT_In-tem_ > 211 s	2/7 (29%)	2/4 (50%)	0.576
CFT_In-tem_ > 130 s	0/7	3/4 (75%)	0.024
MCF_In-tem_ < 57 mm	0/7	3/4 (75%)	0.024
alpha_In-tem_ > 58°	0/7	2/4 (50%)	0.109
ML_In-tem_ > 5%	1/7 (14%)	1/4 (25%)	1.000
MCE_In-tem_ < 108	0/7	3/4 (75%)	0.024
CT_Fib-tem_ > 112 s	1/8 (13%)	7/9 (78%)	0.015
MCF_Fib-tem_ 0 mm	0/9	7/9 (78%)	0.002
Treatment			
Plasma transfusion	0/9	4/9 (44%)	0.082
Tranexamic acid	0/9	5/9 (56%)	0.029

aPTT, activated partial thromboplastin time; CT, clotting time; CFT, clot formation time; AUC, area under the curve; CI, confidence interval; CT, clotting time; CFT, clot formation time; MCF, maximum clot firmness; ML, maximum lysis; PT, prothrombin time; MCE, maximum clot elasticity; MCF, maximum clot firmness; ML, maximum lysis; PT, prothrombin time.

**Table 5:** AUROC analysis for specific factors to predict hyperfibrinolysis in 18 dogs with *A. vasorum* infection

Parameter	AUC	95% CI	*p*
CT_Ex-tem_	0.781	0.552–1.000	0.024
CFT_Ex-tem_	0.951	0.874–1.000	<0.001
PT	0.808	0.593–1.000	0.064
Fibrinogen Clauss	0.336	0.000–0.702	0.321
MCF_Ex-tem_	0.042	0.000–0.115	<0.001
ML_Ex-tem_	0.125	0.000–0.273	0.003
Platelet count	0.250	0.050–0.450	0.072

AUC, area under the curve; CI, confidence interval; CT, clotting time; CFT, clot formation time; MCF, maximum clot firmness; ML, maximum lysis; PT, prothrombin time.

## Data Availability

The data presented in this study is available on request from the corresponding author.
